# Comparison of Radiofrequency Ablation Versus Cryoablation For T1 Renal Tumors: An Evidence-Based Analysis of Comparative Outcomes

**DOI:** 10.3389/fonc.2022.802437

**Published:** 2022-04-22

**Authors:** Hongjin Shi, Jinze Li, Zhinan Fan, Jing Yang, Shi Fu, Haifeng Wang, Jiansong Wang, Jinsong Zhang

**Affiliations:** ^1^ Department of Urology, The Second Affiliated Hospital, Kunming Medical University, Kunming, China; ^2^ Department of Urology/Institute of Urology, West China Hospital, Sichuan University, Chengdu, China

**Keywords:** renal tumor, radiofrequency ablation, cryoablation, outcomes, meta-analysis

## Abstract

**Objective:**

To discuss the differences in the effectiveness and security for T1 renal tumors by radiofrequency ablation (RFA) and cryoablation (CA).

**Methods:**

We systematically searched the Cochrane Library, PubMed, Embase, CNKI databases, and Science databases, and the date was from the above database establishment to August 2021. Controlled trials on RFA and CA for T1 renal tumors were included. The meta-analysis was conducted with the Review Manager 5.4 software.

**Results:**

A total of ten studies with 2,367 patients were included in the analysis. There were no significant differences in complications (odds ratio [OR], 1.23; 95% CI, 0.80 to 1.90; p=0.35), primary technique efficacy rate (OR, 1.01; 95% CI, 0.33 to 3.14; p=0.98), changes in serum creatinine (weighted mean difference [WMD], 0.53; 95% CI, -0.50 to 1.57; p=0.31), or 5-year survival rate (hazard ratio [HR], 1.11; 95% CI, 0.41 to 3.00; p=0.84) among patients undergoing RFA and CA. However, compared with patients who underwent RFA, patients who underwent CA had a lower Local recurrence (OR: 2.25; 95% CI: 1.38 to 3.67; p = 0.001).

**Conclusion:**

The analysis demonstrated that in the treatment of T1 renal tumors, CA may be associated with lower local recurrence rates. However, no differences were observed in terms of primary technique efficacy rate, 5-year survival rate, changes in serum creatinine, and complication rate between groups.

**Systematic Review Registration:**

[https://www.crd.york.ac.uk/PROSPERO/], identifier PROSPERO (CRD42021295160).

## Introduction

Renal cell carcinoma (RCC) is among the top 10 most frequently diagnosed cancers worldwide (1), with an estimated 403,262 new cases and 175,098 associated deaths worldwide in 2018 (2). For patients with localized (cT1) renal masses warranting treatment, multiple guidelines emphasize the use of nephron-sparing treatment (3, 4). According to the American Urological Association (AUA) recommendations, partial nephrectomy (PN) was chosen as the therapy of choice for small renal masses since several studies demonstrated tumor control comparable to radical nephrectomy (3, 5–8). However, due to coexisting disease, renal insufficiency, and advanced age, some patients require less invasive procedures.

Over the last several decades, advances in medical science have expanded the use of surgical and radiological procedures. Because patients with localized renal cell cancers have a high survival rate, minimally invasive ablative treatments have garnered a lot of attention as prospective therapeutic alternatives. RFA and CA are the most often used techniques (9, 10). Compared with excisional surgery, RFA and CA are less invasive (11). Local ablative therapies are repeatable and simple to apply. Psutka et al. (12) observed durable local control of low-risk T1A renal cell carcinoma by RFA. In addition, Thompson et al. (13) observed that in the short-term follow-up, there was no statistically significant difference in the local recurrence survival rate and the metastasis-free survival rate of PN, RFA, and CA for cT1a renal masses. Kunkel et al. (14, 15) published two landmark meta-analyses in 2008, the results suggested that considerable differences in local recurrence rate between RFA and CA. RFA and CA for T1 renal cell tumors are still contentious, leaving clinicians to rely on experience and judgment when deciding on a surgical alternative.

Therefore, we conducted a systematic review and meta-analysis of the efficacy and safety of RFA and CA for the treatment of T1 renal cell tumors, to provide a better clinical reference.

## Materials and Methods

### Search Strategy

We systematically searched the Cochrane Library, PubMed, Embase, CNKI databases, and Science databases, and the date was from the above database establishment to August 2021. We used the following search terms: “kidney neoplasms”, “renal tumor”, “cancer of the Kidney”, “radiofrequency ablation”, “radio-frequency ablation”, “cryoablation”, and “cryosurgery”. Search strategies were tailored for the different search engines. Manual retrieval from the references of subject-related articles was performed to broaden the search. The search was not limited by region or language. Each included study was evaluated independently by two reviewers (H.S. and J.L.), and any differences were resolved by consensus.

### Inclusion/Exclusion Criteria

Following the PICOS principle, studies meeting the following inclusion criteria were admitted: (1) studies performed in adults diagnosed with renal tumor; (2) included patients who received RFA or CA treatment; (3) studies comparing RFA with CA; (4) full papers containing at least one outcome parameters, such as primary technique efficacy rate, changes in serum creatinine, 5-year survival rate, local recurrence and complications after ablation; (5) study type was a randomized controlled trial, cohort study, or case-control study; The following studies were excluded: reviews, case reports, letters, low-quality researches and researches with no detailed data.

### Data Extraction

The following data from each study would be extracted into our meta-analysis: lead author, publication date, study type, study country, study interval, sample size, surgical procedure, primary technique efficacy rate, changes in serum creatinine, local recurrence after ablation, and complications; When continuous variables reported as other forms in the main literature, we calculated the mean and standard deviation (16)

### Quality Assessment

The risk of bias in non-randomized studies of interventions (ROBINS-I) (17) was used to evaluate the literature quality. The literatures included in this study were retrospective controlled studies that met the criteria for the use of seven bias risk measurement tools in ROBINS-I, scoring bias at different stages before, during, and after intervention (low risk, moderate risk, serious risk, critical risk, and no information). Two reviewers (H.S. and J.L.) completed quality evaluation and disagreements were settled by discussion.

### Statistical Analysis

In this study, the Cochrane Collaborative RevMan5.4 software was applied for meta-analysis statistical processing. The weighted mean differences (WMDs) and odds ratio (OR) were calculated for continuous and dichotomous variables, respectively, with 95% confidence intervals (CIs). For oncologic outcomes, including overall survival (OS), hazard ratio (HR) and 95% CI were applied. χ^2^ test and I^2^ test were used to analyze the heterogeneity between the studies. A random-effects model was employed if there was considerable heterogeneity (p < 0.05 or I^2^ > 50%), otherwise a fixed-effects model was used. The Z test was conducted to determine all merged effects, and statistical significance was defined as p < 0.05.

### Registration

The study was registered on PROSPERO (CRD42021295160).

## Results

### Study Characteristics

**Figure 1:**
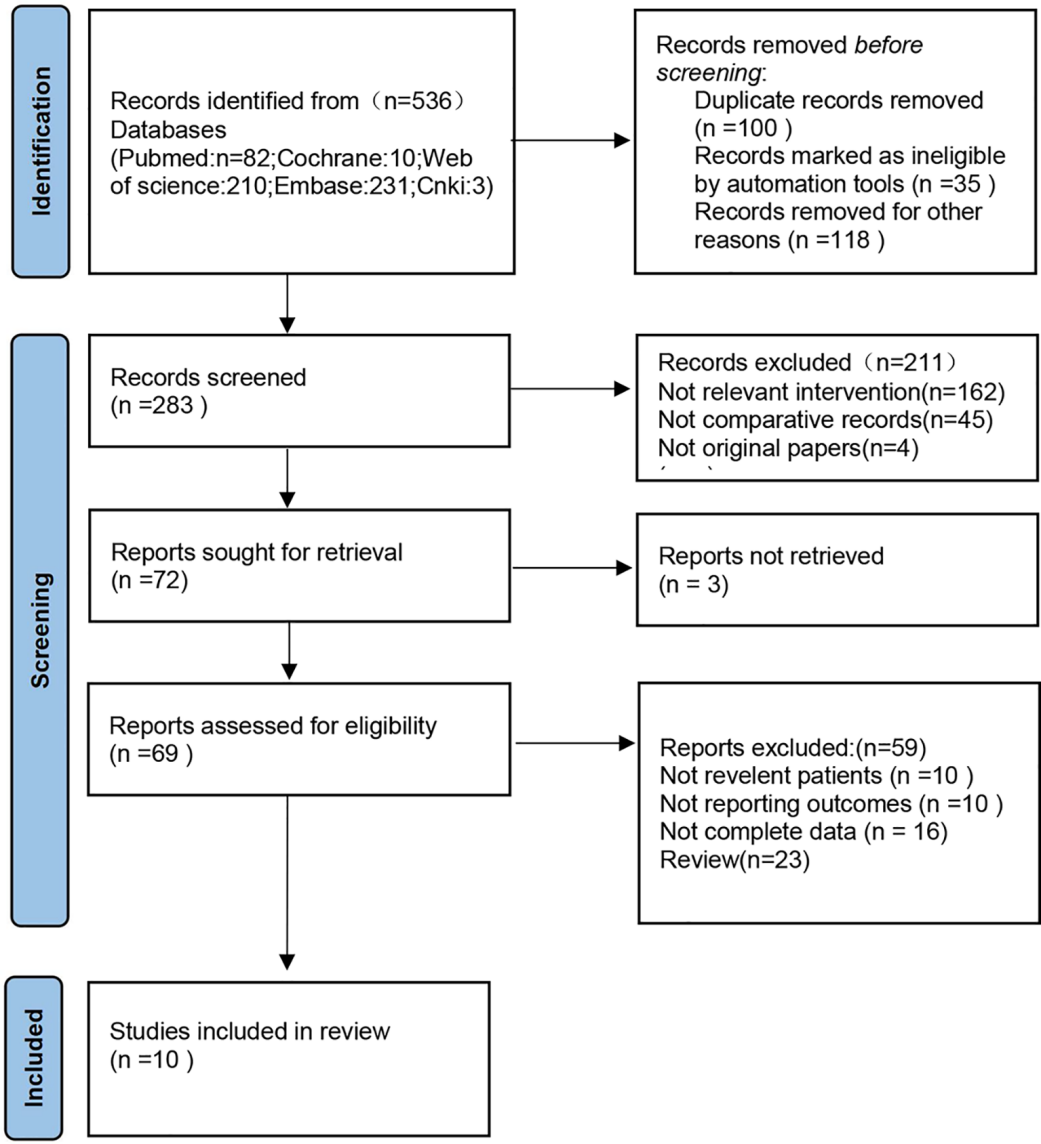
Flow diagram of studies identified, included and excluded.

**Figure 2:**
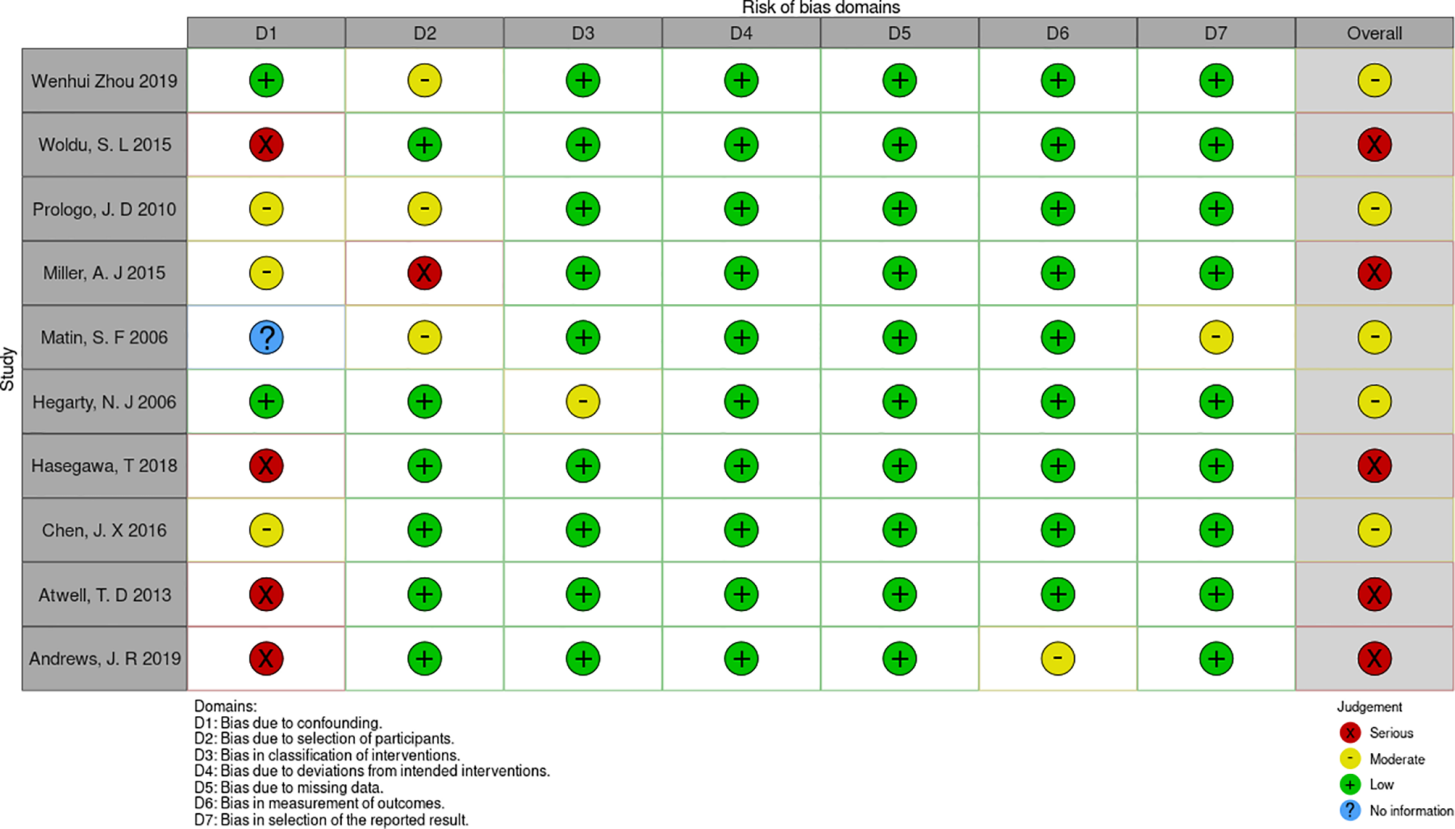
the risk of bias assessment for each trial using the non-randomized studies of interventions (ROBINS-I).

Preliminarily, 536 related articles were identified. 253 of them were eliminated due to duplication or because they were unrelated to our inclusion criteria dependent on the screening records. Following an examination of the whole text, 273 records were deleted. Finally, the remaining ten studies with a total of 2367 patients (1336 in the RFA groups and 1031 in the CA groups) were included in our meta-analysis (18–27) (**Figure 1**). The characteristics of all the included studies are presented in **Table 1**. The seven deviation risk measurement tools of the risk of bias in non-randomized studies of interventions (ROBINS-I) (17) were used to evaluate the literature quality of the ten included studies. There was a certain risk of bias in all studies, most of which were moderate, and the average quality of each study was good. The results are shown in **Figure 2**.

**Table 1:** Baseline characteristics of include studies and methodological assessment.

Studies	Interval	Study design	Intervention	Patients (n)	Tumor size (cm)	BMI (kg/m2)	Age (years)	Follow-up (months)
Zhou et al. (26)	October 2006 to December 2016	Retrospective	RFA/CA	244/26	2.4±0.875/ 2.4±0.65	30 ±9.5/ 31±7.25	73 ±18.5 /68±12	24
Woldu et al. (18)	2007 to 2013	Retrospective	RFA/CA	30/30	2.3 ± 0.6/ 2.1 ± 0.7	31.4 ± 8.8/ 26.8 ± 5.9	69.0 ± 10.6 / 64.2 ± 15.1	NA
Pirasteh et al. (20)	2006 to 2009	Retrospective	RFA/CA	41/70	0.2 (0.8~4.8)	NA	70 (31-91)	NA
Miller et al. (23)	June 2001 to May 2012	Retrospective	RFA/CA	44/61	2.3±0.8/ 3.0 ± 0.7	NA	84.5 ± 3.2 / 83.7 ± 2.9	34.8
Matin et al. (27)	NA	Retrospective	RFA/CA	410/206	NA	NA	NA	24.2
Hegarty et al. (24)	1997 to 2005	Retrospective	RFA/CA	72/161	2.51±0.94/ 2.56±0.90	29.9±8.2/ 29.3±11.15	66.6±12.75/66.3±15	13/39
Hasegawa et al. (25)	March 2006 to October 2014	Retrospective	RFA/CA	23/23	4.94±0.74/ 5.01±0.73	NA	69±12.27/64.17±14.02	32.8/23
Chen et al. (19)	January2004 to January 2014	Retrospective	RFA/CA	70/104	3.2±1.6/ 3.0±1.4	NA	70.8±13/70±11	36/33
Atwell et al. (22)	2000 to 2010	Retrospective	RFA/CA	222/163	1.9±0.5/ 2.3±0.5	NA	68.8±11.6/68.2±11.3	38.4/21.6
Andrews et al. (21)	2000 to 2011	Retrospective	RFA/CA	180/187	1.93±0.20/ 2.84±0.20	NA	71.33±2.76/72±2.74	90/75.6

RFA, radiofrequency ablation; CA, cryoablation; BMI, body mass index; X±Y, NA, not available; NR, not report; mean±standard deviation; X (Y-Z): mean (range).

### Demographic Variables

Demographic variables were analyzed according to the literature included for each outcome parameter, and age was statistically different for the included outcome parameters of changes in serum creatinine (WMD, 4.90; 95% CI, 1.32, 8.49; p=0.007), local recurrence (WMD, -0.57; 95% CI, -1.12, -0.03; p=0.04), and 5-year survival (WMD, -0.61; 95% CI, -1.16, -0.05; p=0.03). We also found statistical differences in tumor size in the literature that included local recurrence(WMD, -0.50; 95% CI, -0.94, -0.05; p=0.03; **Table 2**).

**Table 2:** The demographics of the studies.

Outcome	Variable	Model	WMD or OR(95% CI)	p value	I^2^
Complications	age	Fixed	0.94 [-0.02, 1.90]	p = 0.05	0%
	Sex	Fixed	0.98 [0.75, 1.28]	p = 0.90	3%
	Tumor size	Random	-0.20 [-0.44, 0.04]	p = 0.10	80%
The Primary Technique Efficacy Rate	age	Fixed	0.99 [-0.01, 1.98]	p = 0.05	0%
	Sex	Fixed	1.04 [0.78, 1.40]	p = 0.79	9%
	Tumor size	Random	-0.23 [-0.50, 0.04]	p = 0.10	80%
Changes In Serum Creatinine	age	Fixed	4.90 [1.32, 8.49]	p = 0.007	0%
	Sex	Fixed	0.93 [0.51, 1.70]	p = 0.082	0%
	Tumor size	Fixed	0.05 [-0.14, 0.24]	p = 0.59	0%
Local Recurrence	age	Fixed	-0.57 [-1.12, -0.03]	p = 0.04	34%
	Sex	Fixed	0.90 [0.67, 1.21]	p = 0.47	0%
	Tumor size	Random	-0.50 [-0.94, -0.05]	p = 0.03	98%
5-year Survival Rate	age	Fixed	-0.61 [-1.16, -0.05]	p = 0.03	22%
	Sex	Fixed	1.07 [0.76, 1.52]	p = 0.70	31%
	Tumor size	Random	-0.28 [-1.07, 0.50]	p = 0.48	95%

RFA, radiofrequency ablation; CA, cryoablation; WMD, weighted mean difference; OR, odds ratio; CI, confidence interval.

### Complications

**Figure 3:**
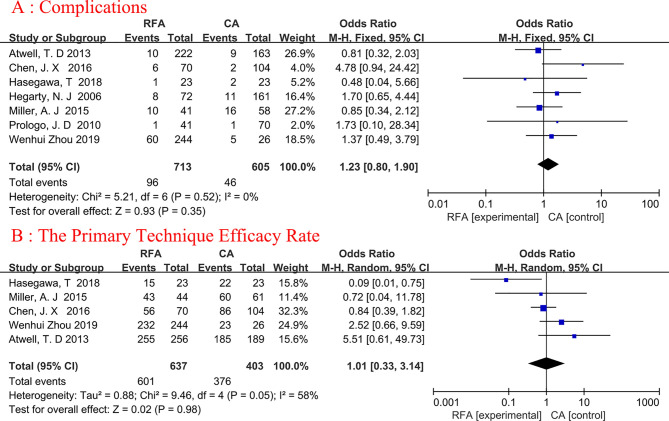
Forest plot and meta-analysis of complications **(A)** and the primary technique efficacy rate **(B)**.

Seven articles were analyzed (19, 20, 22–26). A total of 1318 patients were involved in the study, with 713 undergoing RFA and 605 undergoing CA (**Figure 3A**). The complications were similar between the two groups, and no heterogeneity was discovered. (fixed-effects model: OR, 1.23; 95% CI, 0.80, 1.90; p=0.35; I^2 ^= 0%).

### The Primary Technique Efficacy Rate

A total of five studies on primary technique efficacy rate were conducted (19, 22, 23, 25, 26), The percentage of tumors entirely treated by the original treatment was defined as primary efficacy. Because the heterogeneity was considerable (I^2 ^= 58%), we used a random-effects model (**Figure 3B**). The final results showed no statistical significance between the two ablation techniques (random-effects model: OR, 1.01; 95% CI, 0.33, 3.14; p=0.98; I2 = 58%).

### Changes In Serum Creatinine

**Figure 4:**
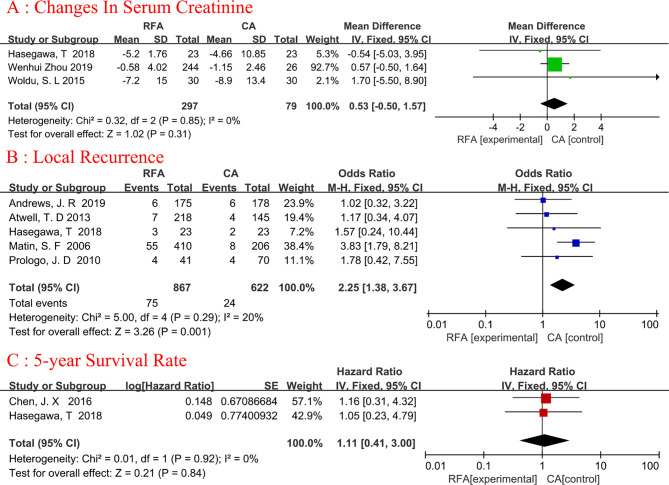
Forest plot and meta-analysis of changes in serum creatinine **(A)**, local recurrence **(B)**, and 5-year survival rate **(C)**.

Three articles were analyzed (18, 25, 26). Serum creatinine changes refer to the comparison of creatinine changes before and after ablation. A total of 376 patients were included, of whom 297 underwent RFA and 79 underwent CA (**Figure 4A**). The changes in serum creatinine were similar between the two groups, and no heterogeneity was found (fixed-effects model: WMD, 0.53; 95% CI, -0.50, 1.57; p=0.31; I^2^ = 0%).

### Local Recurrence

Five studies (19, 20, 22, 25, 27) with 1489 patients (867 in the RFA group and 622 in the CA group) were used to obtain data on local recurrence (**Figure 4B**). The pooled result showed that the CA group had a lower rate of local recurrence than the RFA group(fixed-effects model: OR, 2.25; 95% CI, 1.38, 3.67; p=0.001; I^2^= 20%). The difference in local recurrence between the RFA and CA groups was statistically significant.

### 5-Year Survival Rate

There were 220 patients analyzed in two studies (19, 25). The 5-year survival rate was reported in 90.32% (84/93) of patients who underwent RFA and in 94.49% (120/127) of patients who underwent CA (**Figure 4C**). Meta-analysis demonstrated that RFA offers a comparable 5-year survival rate to CA (fixed-effects model: HR, 1.11; 95% CI, 0.41, 3.00; p=0.84; I^2^ = 0%).

### Subgroup Analysis

**Figure 5:**
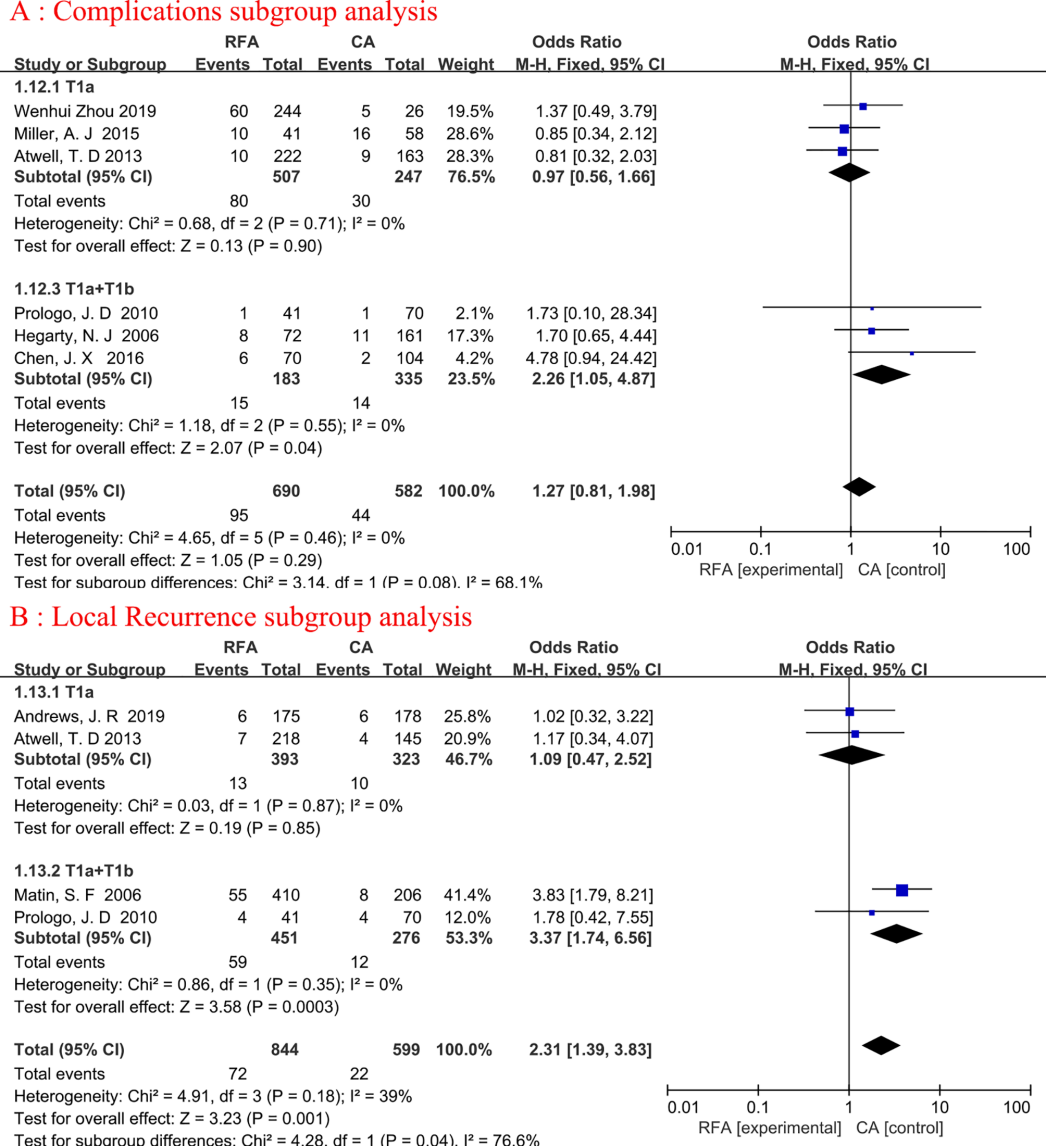
Forest plot and meta-analysis of complications subgroup analysis **(A)** and local recurrence subgroup analysis **(B)**.

Subgroup analyses were conducted for complications and local recurrence based on Tumor staging. In the T1a+T1b tumor staging subgroup, compared with CA, RFA may be associated with higher ablation complications (fixed-effects model: OR, 2.26; 95% CI, 1.05, 4.87; p=0.04; I^2 ^= 0%) and local tumor recurrence (fixed-effects model: OR, 3.37; 95% CI, 1.74, 6.56; p=0.0003; I^2^ = 0%). Detailed data are presented in **Figure 5**.

### Publication Bias

**Figure 6:**
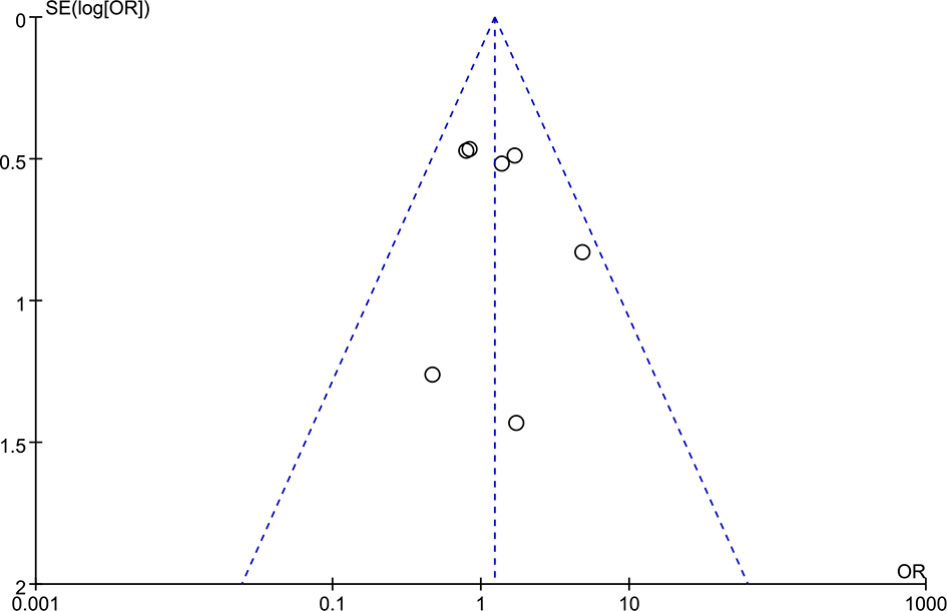
Funnel plot.

The funnel plot was used to evaluate the publication bias of the studies, and the results showed that the distribution of each study was roughly conical, but there was still some publication bias (**Figure 6**).

## Discussion

For patients with limited (T1) renal masses requiring treatment, several guidelines emphasize the use of treatment with preservation of the renal unit, partial nephrectomy emerged as the treatment of choice for small renal masses (3, 4). Some patients cannot tolerate the surgical trauma and perioperative complications caused by radical nephrectomy or partial nephrectomy. minimally invasive ablative therapies have received much attention as potential treatment options. The most popular approaches are RFA and CA (9, 10).

RFA and CA were initially applied in the treatment of clinical T1a renal cancer and showed good clinical results (3, 28–31). The indications for ablation have later been expanded to include larger renal tumors (12). Several studies have shown that RFA and CA for clinical T1b renal cancer can provide oncologic efficacy comparable to nephrectomy and better protection of renal function (32, 33). The superiority of one approach over the other in the ablative treatment of patients with T1 kidney cancer remains uncertain, and most of the previous relevant studies are retrospective, and the limited number of patients and the single-center nature of these studies row not be sufficiently powered to demonstrate the superiority between the two approaches. For these reasons, we performed a meta-analysis to discuss the differences in safety and efficacy of the two surgical approaches for the treatment of T1 renal tumors.

Complications are a significant factor for estimating the safety of ablation procedures. In terms of complications, our study indicated that there was no significant statistical difference between the RFA and CA groups, which was consistent with most studies. Common complications of ablation include procedural bleeding, perirenal hematoma, and transient hematuria (34). Visceral injury or damage to the collection system may also occur. Bleeding is the most common complication. Bleeding is usually caused by direct mechanical vascular injury caused by the probe. CA may cause massive bleeding after the frozen probe is removed or the ice ball ruptures. However, the results of the study conducted by Johnson et al. (35) suggested that the two methods had significant differences in complications, and the results suggested that the complication rate of RFA was higher than that of cryotherapy. In Professor Johnson’s research, we found that the main reason may be the immature technology and imperfect equipment of RFA and CA (2004). In addition, The size and location of kidney tumors are also related to interfering factors (36).

In terms of the primary technique efficacy rate, the results of the meta-analysis showed that there was no significant difference between RFA and CA. However, Professor Hasegawa et al. (25) found that the primary technique efficacy in the CA group was significantly higher than that in the RFA group. (65% vs. 96%, p = 0.02). After reviewing the literature, we found that the patient data came from 3 different institutions. The main reason for this difference in results may be the different experiences of doctors in different institutions. In addition, the number of ablation needles also affects the success rate of an operation. The main technique for ablation using a multi-needle system is more effective than a single needle. Hasegawa et al. (25) found that the CA group used significantly more needles. Indeed, multiple needles might be easily used for CA, but RFA required some additional systems. More prospective studies are needed for further verification.

The changes in serum creatinine before and after ablation, our meta-analysis did not show specific differences between the two procedures. Quite a several studies have compared the renal function protection of RFA and CA, and the results showed that there was no difference in renal function between RFA and CA (32, 33, 37), and no patients need hemodialysis after ablation treatment, and the analysis of each included study was highly consistent. In addition, Turna et al. (38) reviewed their institutional experience in treating patients with isolated kidneys through various nephron preservation procedures and their research found that compared with CA or RFA, renal function damage was more obvious after partial nephrectomy. We speculate that the remaining renal parenchymal compensatory ability is weakened and there is no compensation from the contralateral kidney in patients with a solitary kidney after partial nephrectomy. It can be seen that for patients with a solitary kidney, RFA and CA may be better than partial nephrectomy in terms of renal function protection for T1 renal tumors.

As one of the indicators to measure the effectiveness of ablation methods, local recurrence has always been the focus of attention of clinicians. In our meta-analysis, the local recurrence rate of CA was lower than that of RFA. Kunkel et al. (14, 15) published two landmark meta-analyses in 2008 and found that RFA and CA had a considerable difference in the rate of local tumor recurrence. Specifically, 12% of RFA and 5% of CA procedures reported local recurrence, further confirming our results. The results of our subgroup analysis showed that there was no statistical difference in local recurrence in T1a stage renal cancer. When the included patients included T1a and T1b patients, there was a significant statistical difference in local recurrence. This may have a certain relationship with the size of the tumor, Gervais et al. (39) found that RFA was 100% effective in treating kidney tumors of 3 cm or smaller, and the effective rate was 81% in treating tumors larger than 3 cm. Similarly, Zagoria et al. (40) achieved a 100% local control rate in 95 RCCs with a diameter of 3.5 cm or smaller, but only 47% of the RFA treatments in 30 RCCs with a diameter of 3.6 cm or larger. This group observed that for every 1 cm increase in tumor size, the risk of treatment failure doubled. CA was effective for larger kidney tumors, especially those with a maximum diameter of more than 3 cm (41). This can be attributed to the synergistic effect of the cryoprobe in producing ice balls large enough to enclose tumors of 8 cm in size, and the ability to effectively monitor the ice balls because it contains the tumor.

In the present analysis, only two studies compared the 5-year survival rate of the two groups (RFA and CA). The meta-analysis showed that RFA provides a 5-year survival rate comparable to CA. These results are not only consistent with previous single-arm studies of RFA and CA (13, 42), but further, suggest that two ablation modalities can provide similar therapeutic outcomes. However, due to the limited literature and sample size, it is necessary to conduct long-term follow-up studies to determine the potential difference in efficacy between the RFA group and the CA group.

We completed this meta-analysis under the strict guidance of PRISMA (43), but there are still some limitations. First of all, the included studies adopt the retrospective design, and some of the studies have very small sample sizes. This means that they are not persuasive and the level of evidence is low. Second, the number of clinical studies included in the evaluation of various indicators is limited, so it is difficult to obtain effective evidence. Third, many studies report shorter follow-up periods, and only three studies report a 5-year survival rate. Fourth, most literatures relating to long-term survival outcomes are not included because there are few studies directly comparing RFA and CA. As a consequence, network meta-analysis can better compare the long-term outcomes of RFA and CA on T1 RCC in this situation. Nevertheless, our meta-analysis has high evidence, and most of the included studies were published in the past five years. The analysis of the outcome indicators is comprehensive, which significantly improves the credibility of our results. In addition, since RFA and CA are primarily recommended for patients who are not candidates for surgery, it is important for clinicians to consider non-cancer-related deaths when making clinical decisions.

## Conclusion

Our analysis showed CA may be associated with lower local recurrence rates. However, there were no significant differences between RFA and CA in complications, primary technical success, changes in serum creatinine, and 5-year survival rate. More studies are still required to support our conclusion.

## Data Availability Statement

The original contributions presented in the study are included in the article/supplementary material. Further inquiries can be directed to the corresponding author.

## Author Contributions

JZ and JW: conception and design. HS, JL, Z-NF, and JY: obtain data and critically revise manuscripts of important knowledge content. HS, JL, and Z-NF: analysis and interpretation of data. HS and JL: drafting of the manuscript. JZ: supervision. All authors: read and agree to the published version of the manuscript.

## Funding

Supported by the National Natural Science Foundation of China, No. 81972395 and No.82060464.

## Conflict of Interest

The authors declare that the research was conducted in the absence of any commercial or financial relationships that could be construed as a potential conflict of interest.

## Publisher’s Note

All claims expressed in this article are solely those of the authors and do not necessarily represent those of their affiliated organizations, or those of the publisher, the editors and the reviewers. Any product that may be evaluated in this article, or claim that may be made by its manufacturer, is not guaranteed or endorsed by the publisher.
